# The Effect of Phenazine-1-Carboxylic Acid on the Morphological, Physiological, and Molecular Characteristics of *Phellinus noxius*

**DOI:** 10.3390/molecules21050613

**Published:** 2016-05-11

**Authors:** Huazhi Huang, Longhua Sun, Keke Bi, Guohua Zhong, Meiying Hu

**Affiliations:** 1 Key Laboratory of Natural Pesticide and Chemical Biology, College of Agriculture, South China Agricultural University, Guangzhou 510642, China; scauhhz@163.com (H.H.); guohuazhong@scau.edu.cn (G.Z.); 2 Guangzhou Insitute of Forestry and Landscape Architecture, Guangzhou 510405, China; senlonghua@163.com (L.S.); gzbikeke@163.com (K.B.)

**Keywords:** antifungal activity, phenazine-1-carboxylic acid, *Phellinus noxius*, mycelia morphology, biological characterization, reactive oxygen species, gene expression

## Abstract

In this study, the effect of phenazine-1-carboxylic acid (PCA) on morphological, physiological, and molecular characteristics of *Phellinus noxius* has been investigated, and the potential antifungal mechanism of PCA against *P. noxius* was also explored. The results revealed that PCA showed *in vitro* antifungal potential against *P. noxius* and completely inhibited *P. noxius* hyphae at concentrations >40 μg/mL. PCA inhibited both mycelial growth and the loss of mycelial biomass *in vitro* in a dose-dependent manner. Morphological changes in PCA-treated *P. noxius* hyphae, such as irregularly swollen mycelia as well as short hyphae with increased septation and less branching, were observed by optical microscopy. The intracellular reactive oxygen species (ROS) levels were significantly increased in PCA-treated *P. noxius* cells as compared to control groups. Induced hyperpolarization of the mitochondrial membrane potential (MMP), repressed superoxide dismutase (SOD) activity and up-regulated gene expression of seven tested genes were also found in PCA-treated *P. noxius* groups. Thus, the present results suggested that the mechanism of action of PCA against *P. noxius* might be attributed to direct damage of mycelium and high intracellular ROS production, and indirect induction of genes involved in cell detoxification, oxidation-reduction process, and electron transport of the respiratory chain.

## 1. Introduction

*Phellinus noxius* is the casual agent of brown root rot disease, which can cause slow and reduced growth in trees, discoloration and wilting of leaves, defoliation, and dieback of branches and eventually lead to death [1,2]. The fungus was first reported in Singapore by Corner in 1932 as *Fomes noxius* and reclassified by Cunningham in 1965 as *P. noxius* [3]. It is widely distributed in tropical and sub-tropical regions of Southeast and East Asia, Oceania, Central America, Australia and Africa [2]. According to previous reports, the fungus has a very wide host range and shows little host specificity [1,3,4]. To date, more than 200 agricultural and forest plant species representing 59 families have been recorded as host plants, most of which are woody but some of which are herbaceous plant hosts [3,4]. The fungus may spread from adjacent infected trees through root-to-root contact or from wood debris of dead trees where *P. noxius* can remain viable in the soil for more than ten years, but it is still not clear whether air-borne basidiospores may function to establish new disease foci [1,4]. More recently, *P. noxius* has become a serious problem in urban areas, such as in Australia, Japan, Taiwan, Hongkong, and Macao, where it has killed trees, including a variety of perennial fruit trees, ornamental trees, and ancient tree species [1,4,5]. Therefore, tree health problems have attracted more and more attention in both the public and research realms. Although some efforts were made, and sometimes successfully, to prevent brown root rot disease spreading rapidly, such as removing all the infected trees and as many roots as possible, installing root barriers or ditching around the infected site to reduce the rate of spread, flooding the infested soil, and replanting resistant species in the infested areas [3,6], few reports have been involved in chemical controlling measure. An ammonia-based chemical reagent has been developed to be effective against *P. noxius*, but it has side effects on soil characteristics and root growth [7]. Recently, the chemical fumigant dazomet has been reported to be a possible means of preventing *P. noxius* incursion in agriculture [8]. In fact, there is no effective way to completely eliminate this fungus in soil by the existing chemical strategies. Moreover, synthetic fungicides might not be a good choice in urban areas, where extensive environmental contamination occurs. Nowadays, the biological control of *P. noxius* shows promise due to its environmental friendliness [5].

Naturally, phenazines are a large group of nitrogen-containing heterocyclic compounds, mainly synthesized by *Streptomyces* and *Pseudomonas* species [9], which play major roles in iron acquisition, respiration, redox balancing, signaling, and community development in their producers because of their redox activity [10]. Phenazine-1-carboxylic acid (PCA), an antibiotic that is the main active ingredient of *Pseudomonas* spp., which has a strong suppressive activity towards various plant fungal pathogens, nematodes (*Caenorhabditis elegans*) and Gram-negative bacterium (*Xanthomonas oryzae*) [9,10,11]. In China, PCA has been registered as the biofungicide “Shenqinbactin” because of its high fungicidal efficiency, low toxicity to humans and animals, environmental compatibility, and improvement of crop production [10,12]. In a recent study, the PCA had good antifungal activity against the major part of the 25 phytopathogenic fungi and oomycetes that they were mainly Ascomycetes or Deuteromycota, but one species *Rhizoctonia solani* assayed in that study belonging to Basidiomycota was shown to be highly tolerant to PCA [9]. However, there have been no further studies of how PCA affects those fungal pathogens. To our knowledge, there is no literature available on PCA inhibiting *P. noxius* attacking a variety of tree species. The objective of the present work is to evaluate the *in vitro* antifungal activity and to investigate how PCA affects *P. noxius* by biochemical and molecular methods.

## 2. Results and Discussion

### 2.1. Effects of PCA on Growth and Morphology of P. noxius Mycelium

**Figure 1:**
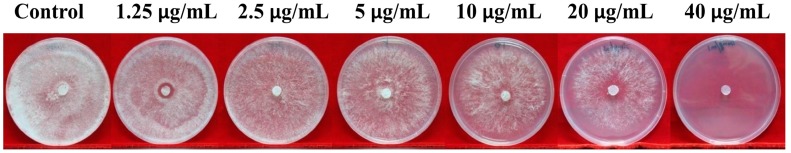
Growth inhibition of *P. noxius* on potato dextrose agar (PDA) plates in the presence or absence of the serial concentration (1.25–40 μg/mL) of phenazine-1-carboxylic acid.

**Figure 2:**
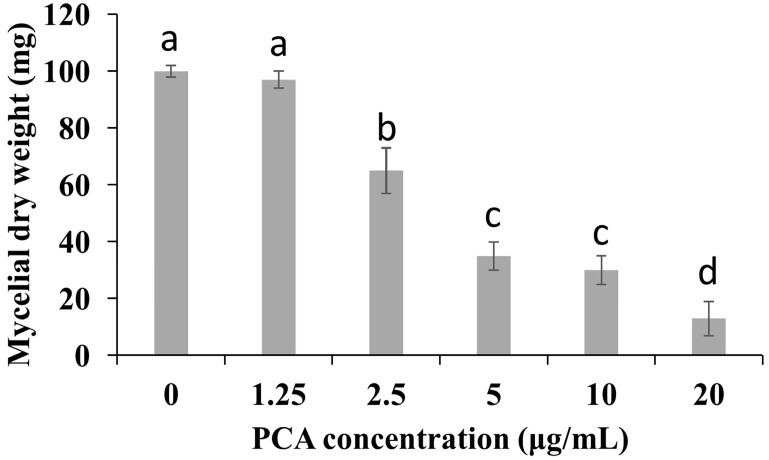
Effect of phenazine-1-carboxylic acid (PCA) on the dry weight of the mycelium of *P. noxius* in the presence or absence of the serial concentration (1.25–20 μg/mL) of PCA. Values were presented as mean ± S.E. Data presented were the means of pooled data (*n* = 6). Values followed by a different lowercase letter are significantly different (*p* < 0.05).

The *in vitro* antifungal activity of PCA against mycelial growth of *P. noxius* was shown in Figure 1. The inhibition of fungal mycelial growth was obvious at 9 days compared to the non-PCA-treated group. PCA was able to completely inhibit the growth of *P. noxius* at 40 μg/mL. This inhibition lasted for at least 30 days. Further details are shown in Figure 2. This fungus exhibited a slower growth rate when grown in potato dextrose broth (PDB) with the serial concentration (1.25–20 μg/mL) of PCA as compared to the non-PCA-treated group; moreover, PCA treatment significantly reduced the growth mycelial biomass. Both the inhibitory efficacy of mycelial growth and the loss of mycelial biomass were positively correlated with the PCA concentration.

**Figure 3:**
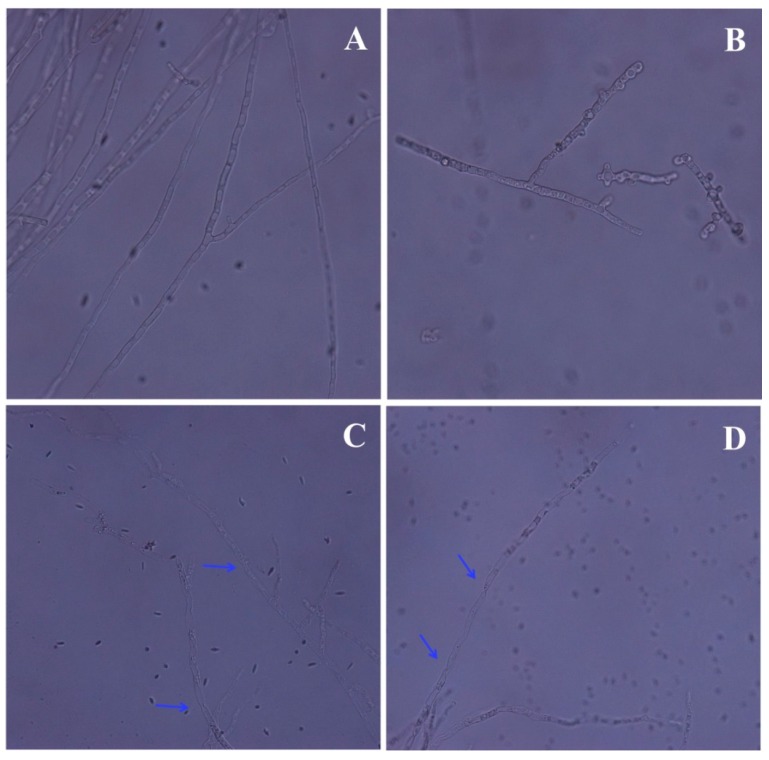
Optical microscopy of *P. noxius* mycelia with or without PCA (40×). (**A**) Control; (**B**) 10 μg/mL; (**C**) 20 μg/mL; (**D**) 40 μg/mL.

**Figure 4:**
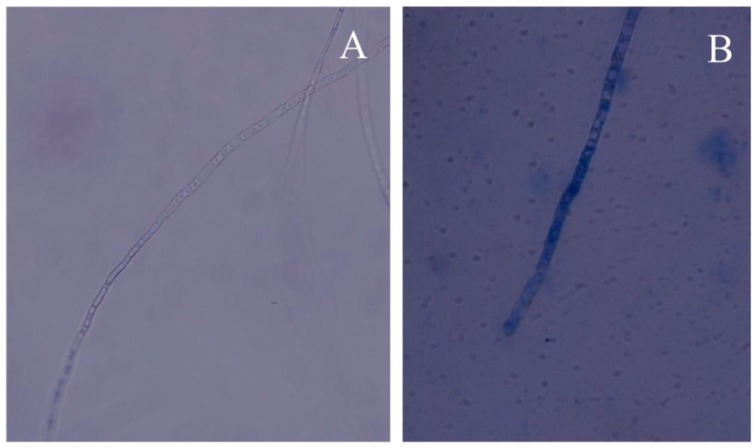
PCA-induced cell death confirmed by 0.02% methylene blue staining (40×). (**A**) Control; (**B**) 20 μg/mL PCA.

Moreover, the hyphal morphological changes were examined via optical microscopy, which indicated that treatment with PCA had a significant effect on mycelia growth (Figure 3). After the PCA treatment, fungal hyphae revealed irregularly swollen mycelia and short hyphae with increased septation and less branching compared to the control intact mycelium with normal branching and septation. In this study, *P. noxius* hyphea treated with PCA were stained with methylene blue to determine viability. PCA-treated hyphae (approximately 30% in 10 μg/mL or 80% in 20 μg/mL) retained the oxidized form of methylene blue because of the loss of the reducing capacity of the dead hyphae. However, the viable hyphae rapidly converted the methylene blue dye to its colorless form, leucomethylene blue, although the hyphae without PCA treatment showed a low level of staining with methylene blue (<3%). Therefore, this finding indicated a fungicidal effect of PCA (Figure 4). Except for abnormal fungal growth caused by PCA (Figure 3B–D), it obviously caused cell lysis or cell death at concentrations above 20 μg/mL.

The present study revealed that PCA had an obvious inhibitory effect on the hyphal growth of *P. noxius*. PCA inhibited both mycelial growth and the loss of mycelial biomass *in vitro* in a dose-dependent manner. It is known that PCA belongs to a group of antibiotic phenazines that is primarily derived from *Pseudomonas* spp. [10,13] and has been reported to possess antimicrobial activity against a variety of phytopathogens such as *Gaeumannomyces graminis* [14], *Seiridium cardinale* [15], *Phytophthora meadii* [16], a Gram-negative bacterium *X.* *oryzae* [10], and a model nematode *C.* *elegans* [11]. Our results are in agreement with these previous studies demonstrating that PCA is characterized by high antimicrobial activity, though they showed a variable sensitivity to PCA, which may be because the different fungi have evolved different strategies to avoid several phenazine compounds [9,10]. In this study, we noticed the hyphae shape in most cells was completely altered by PCA, and lysis of hyphae occurred when exposed to PCA at concentrations above 20 μg/mL. The similar lytic phenomenon of PCA was found when the zoospore suspensions of *P. meadii* were treated with PCA [16]. Some carboxylic acid-functionalized small molecules, such as a polyunsaturated long fatty acid—docosahexaenoic acid—and medium-chain saturated fatty acids and related compounds, were indicated as having fundamental effects on cell membrane by destabilizing lipid bilayers [17,18], but membrane-lytic effects of PCA on *P. noxius* remain unclear.

### 2.2. Mechanism of Action of PCA in P. noxius

**Figure 5:**
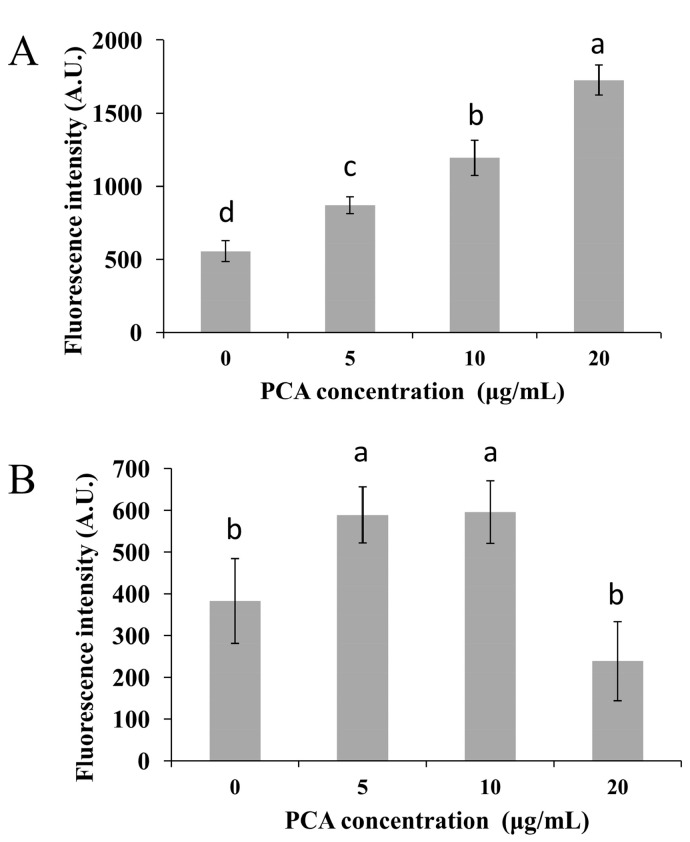
Effects of PCA on intracellular ROS accumulation (**A**) and mitochondrial membrane potential (**B**) in *P. noxius*. Values were presented as mean ± S.E. Data presented were the means of pooled data (*n* = 6). Values followed by a different lowercase letter are significantly different (*p* < 0.05).

It is known that intracellular reactive oxygen species (ROS) are generated in cellular response to exogenous sources such as xenobiotics compounds and pathogen invasion as well as during mitochondrial oxidative metabolism [19]. To examine the intracellular ROS change by PCA, *P. noxius* protoplast cells were treated with PCA (0–20 μg/mL) for 1 h, and ROS levels were measured by using the cell-permeable substrate 2’,7’-dichlorodihydrofluorescein diacetate (DCFH-DA). As shown in Figure 5A, the intracellular ROS accumulation in *P. noxius* was obviously affected by PCA. We found that the ROS product differed in the presence of PCA (0–20 μg/mL) and resulted in a significant increase in the intracellular fluorescence when compared to the control, indicating a higher ROS production in the group treated with PCA. The relative intensity of fluorescence as measured by fluorescence microplate reader increased from 556 arbitrary units (AUs) for cells in the absence of PCA to 1726 AUs in the presence of 20 μg/mL of PCA. Our results indicated that PCA generates ROS in *P. noxius*.

**Figure 6:**
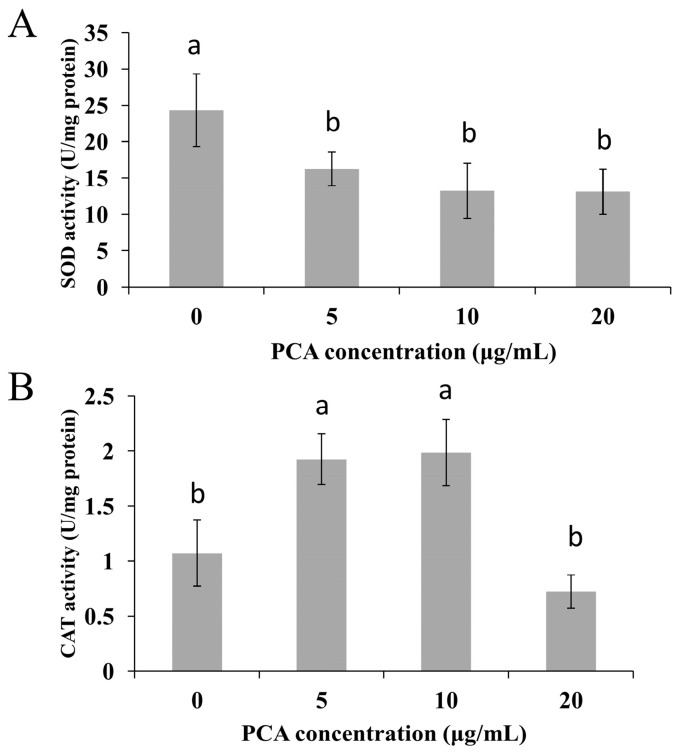
Effects of PCA on SOD (**A**) and CAT (**B**) in *P. noxius*. Values were presented as mean ± S.E. Data presented were the means of pooled data (*n* = 6). Values followed by a different lowercase letter are significantly different (*p* < 0.05).

Mitochondria are considered the main source of ROS in the cell. Rhodamine 123 was used as a probe of mitochondrial membrane potential (MMP or ΔΨm) because of its reliable and sensitive evaluation [20]. As shown in Figure 5A, the presence of PCA (5–10 μg/mL) triggered hyperpolarization of MMP with a 150% increase in the relative fluorescence intensity in the *P. noxius* protoplast as compared to the non-PCA-treated group. However, the MMP at 20 μg/mL PCA was reduced, which could be the result of high sensitivity of protoplast cells when analyzed. Higher MMP was also reported in *Benjaminiella poitrasii* yeast cells treated with phenazine-1-carboxamide, which may lead to more ROS production [21]. In addition, superoxide dismutase (SOD) activities in *P. noxius* were found to be repressed by PCA at 2 h (Figure 6A), and CAT activity was increased by PCA at 2 h, except for the higher concentration of PCA (20 μg/mL) (Figure 6B). The lower SOD and limited CAT activities in the presence of a higher amount of PCA (20 μg/mL) are likely to reduce ROS scavenging ability, which is consistent with a recent report on *X. oryzae* [10]. The results indicated that the higher levels of ROS in *P. noxius* triggered by PCA play important roles in antifungal action. Consistent with many previous reports, similar redox activities have been shown to be important for the antimicrobial activity of other phenazines, such as clofazimine [22], pyocyanin [23,24], 5-methylphenazine-1-carboxylic acid [25], and phenazine-1-carboxamide [21].

### 2.3. Effects of PCA on Gene Expression of P. noxius

**Figure 7:**
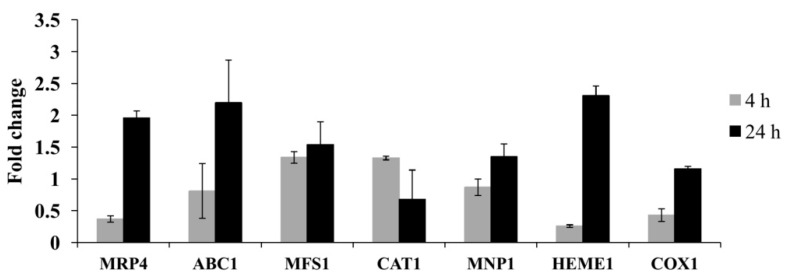
Gene expression profile of seven selected *P. noxius* genes treated with 5 μg/mL PCA for 4 h and 24 h. Relative expression levels in relation to elongation factor 1-alpha expression are calculated from Ct values according to the 2^–ΔΔCt^ method.

To determine if PCA was responsible for the change in gene expression, we selectively analyzed seven genes for qRT-PCR that are involved in cell detoxification, the oxidation-reduction process, and electron transport of the respiratory chain. The results are given in Figure 7; all seven genes were upregulated at all two time points in response to low-level PCA. Interestingly, the expression of genes at 24 h was more upregulated than those at 4 h. It is well known that the ATP-binding cassette (ABC) and the major facilitator superfamily (MFS) transporters can play a major role not only in fungicide sensitivity and resistance but also in the efflux of toxic components and metabolites from the cell [26]. Furthermore, they are involved in a large variety of molecular events such as protein secretion, nutrient uptake, and pathogenesis [27]. In this study, all three selected cellular transport-related genes were upregulated in response to PCA, suggesting that they could be involved in PCA detoxification in order to improve the cell viability.

Peroxidases are heme-containing enzymes that use hydrogen peroxide as the electron acceptor to catalyze a number of oxidative reactions. The expression of manganese peroxidase (MNP) is often induced by a heat shock in nitrogen-limited cultures and regulated by H_2_O_2_ and various chemicals [28]. The MNP1 gene was upregulated by PCA in this study. In addition, the previous study revealed that most fungal species have multiple copies of catalase genes, which might function independently by individual catalases or cooperatively by catalase gene family members [29,30]. Catalases are enzymes responsible for the degradation of H_2_O_2_ and protection of the cell against oxidative stress and ROS accumulation. However, the upregulation of catalases in this study may be the result of a compensatory mechanism for increasing catalase activity, which is inhibited by PCA in an attempt to protect the cell in response to PCA-induced apoptotic cell death effects. Cytochrome c oxidase subunit 1 (COX1) is a gene encoded for the mitochondria-encoded respiratory chain subunit. COX1 was upregulated in response to PCA in this study, which indicated that PCA induces the gene expression in *P. noxius* associated with mitochondrial respiration. Therefore, consistent with previous published data, PCA, like other phenazines, can impact mitochondrial activity [10,31].

## 3. Materials and Methods

### 3.1. Fungal Strain, Culture Conditions, and Chemicals

*Phellinus noxius* was obtained from infected tree roots (*Sterculia lanceolata*) in Guia Hill Municipal Park of Macao. Fungal isolate was stored in sterile tubes containing potato dextrose agar (PDA) and routinely grown on PDA plates. PCA (>98%) was a generous gift from Prof. He Yawen (Shanghai Jiao Tong University) [32]. For addition to cultures, PCA was dissolved in chloroform/methanol (4:1, *v*/*v*) as indicated. All other chemicals and regents are of analytical grade purchased in China.

### 3.2. Effects of Inhibition of PCA in P. noxius on PDA Plate and PDB Medium in Vitro

The sensitivity of *P. noxius* to PCA was assessed in PDA plates. Mycelial discs (5 mm) were removed from one-week-old PDA plates and placed in the center of a 90-mm-diameter petri plate containing PDA plates supplemented with six different concentrations of PCA (1.25, 2.5, 5, 10, 20, and 40 μg/mL), these plates without PCA as control. Each treatment was performed in triplicate. The petri plates were incubated at 30 °C for 9 days, and radial growth was measured at the end of the trial. Growth inhibition of *P. noxius* by PCA was quantified as described previously [9].

For PDB medium, 150 mL conical flasks containing 40 mL of potato dextrose broth (PDB) supplemented with five different concentrations of PCA (1.25, 2.5, 5, 10, and 20 μg/mL) were inoculated with mycelial discs (5 mm), excised from one-week-old PDA plates with chloroform/methanol (0.1%, *v*/*v*) as control. Each treatment was performed in triplicate. Flasks were incubated on a rotary shaker (220 rpm) at 30 °C for 6 days. After 72 h, mycelia were examined under optical microscopy (Nikon DS-Ril-U2, Tokyo, Japan). At the end of the incubation period, mycelia were collected from each flask, dried at 80 °C, and then weighed.

### 3.3. Effect of PCA on Intracellular Reactive Oxygen Species (ROS) Accumulation in P. noxius

Intracellular ROS accumulation was measured with a ROS assay kit (Beyotime, Nantong, China) according to the instruction manual. In brief, Protoplasts from fungal vegetative hyphae were produced as described previously [33] and stabilized in STC medium (1.0 M sorbitol, 10 mM Tris-HCl (pH 8.0), and 50 mM CaCl_2_) to assess PCA effects. Protoplasts were resuspended in a 2′,7′-dichlorodihydrofluorescein diacetate (DCFH-DA) solution (1:1000 diluted with STC medium). The fungal suspensions were then incubated at 30 °C for 20 min in the centrifuge tubes, which were inverted every 5 min. Extracellular DCFH-DA was removed by centrifugation at 6000 rpm for 8 min at room temperature, followed by three washes with STC medium, and the cells were then resuspended in STC medium. The 96-well plates were treated with PCA (5–20 μg/mL) and chloroform/methanol (0.2%, *v*/*v*) as control, and 1 ×  10^4^ cells were then added to each well. Each assay contained triplicates for each concentration. The oxidation of DCFH by ROS was determined by measuring the mean fluorescent intensity of DCFH by a Synergy H1 Microplate reader (Biotek, Winooski, VT, USA) at 488 nm excitation/525 nm emission.

### 3.4. Effect of PCA on Mitochondrial Membrane Potential (MMP)

The MMP assay was performed essentially as described in [21], and Protoplast cells (1 × 10^6^ cells/mL) were suspended in STC medium containing different concentrations of PCA (5–20 μg/mL) and kept at 30 °C for 120 min. After 120 min, 1 μM Rhodamine 123 was added and further incubated for 30 min. Each assay contained triplicates for each concentration. The fluorescence intensities were measured by a Synergy H1 Microplate reader (Biotek) at 480 nm excitation/525 nm emission.

### 3.5. Effects of PCA on Activities of Superoxide Dismutase (SOD) and Catalase (CAT) in P. noxius

*P. noxius* was grown in yeast peptone dextrose (YPD) medium for two days, which was then diluted 20 times in fresh YPD medium supplemented with different concentrations of PCA (5–20 μg/mL) and chloroform/methanol (0.1%, *v*/*v*) as control. Fungal mycelia were collected after incubation at 30 °C for 2 h. Each assay contained triplicates for each concentration. Enzyme activities of SOD and CAT were assayed and calculated with a SOD assay kit (Total SOD) and CAT assay kit as previously described [10].

### 3.6. Quantitative Real-Time PCR

Mycelial discs (5 mm) excised from one-week-old PDA plates were used to inoculate 50 mL of PDB in 150-mL conical flasks. The flasks were incubated at 30 °C/250 rpm for two days. Mycelia were aseptically transferred to 200 mL fresh PDB medium in the presence or absence of 5 μg/mL PCA. Mycelia were harvested by filtration through two-layer cheesecloth after 4 h and 24 h, washed thoroughly with sterile water, quickly frozen in liquid nitrogen, and disrupted by grinding, and total RNA was extracted with TRIzol reagent (Invitrogen) and reverse transcribed into cDNA with ReverTra Ace qPCR RT Master Mix with gDNA Remover (Toyobo life science, Shanghai, China) according to the instruction manual. Seven genes were analyzed, including three cellular transport-related genes [multidrug resistance protein 4 (MRP4), a ATP-binding cassette protein (ABC1) and a MFS transporter (MFS1)], three peroxidase genes (catalase (CAT1), manganase peroxidase 1 (MNP1) and a heme peroxidase (HEME1)), a component of the mitochondrial electron transport chain (cytochrome c oxidase subunit 1, COX1), and elongation factor 1-alpha (EF1α) was used as reference gene. The qRT-PCR reactions were performed using SYBR Premix Ex Taq (Perfect Real Time) (Takara, Dalian, China) and the Thermal Cycler Dice Real Time System (Takara) according to the manufacturer′s protocol. Primers used in this study are listed in Table 1. The relative changes in the gene expression were quantified using the 2^–ΔΔCt^ method [34].

### 3.7. Statistical Analysis

A completely randomized design was used for all treatments. Data were presented as mean ± standard error (SE). The mean separations were carried out using Duncan′s multiple range tests, and significance was determined at 5% level (SPSS Version 19.0, IBM, New York, NY, USA) [35].

## 4. Conclusions

In the current study, it demonstrated that PCA could significantly inhibit the mycelial growth of *P. noxius* *in vitro* in a dose-dependent manner. PCA-treated fungus showed the arrested growth or eventual cell death, as evidenced by the release of a low level of SOD, the production of reactive oxygen species, high mitochondrial membrane potential, and morphological characteristics. At the molecular level, to our knowledge, this is the first study of gene expression for *P. noxius* in response to an antifungal agent PCA. Our results indicated that PCA has a broad spectrum of cellular effects. Due to its innate inhibition potential, PCA may be used as a potent antifungal agent to control *P. noxius* that attacks a variety of tree species.

## Figures and Tables

**Table 1:** Oligonucleotide primers used for qRT-PCR.

Gene Name	Putative Function	Sequence (5′–3′) ^1^	Amplicon (bp.)
MRP4	multidrug resistance protein 4	GGGTTTGACCTCTATCCGAA (F)	105
		CCAGCAACATCGACCAATAC (R)	
ABC1	ATP-binding cassette protein	AGTGCTCGTGAAATGAAACG (F)	96
		ACTCCGAATGGTGGCTAATC (R)	
MFS1	MFS transporter	CTCCGGTTCAACATCACATC (F)	105
		AAGGGTCATCTGGTCCATTC (R)	
CAT1	catalase	CCTTACGATTCTCCACCGTT (F)	107
		CCAATCAAGATTTCCGTCCT (R)	
MNP1	manganese peroxidase 1	TCTACGATCTTGCTGATGCC (F)	91
		TCGTGGAAAGTGAGACGAAG (R)	
HEME1	heme peroxidase	ATCGGATCAAATGGGTTCAT (F)	98
		GGCCATTTCATGGCTCTTAT (R)	
COX1	cytochrome c oxidase subunit 1	TTTAACCTTGGTTTCACTGATGA (F)	85
		GGTCACTGATAGGTGGCTGA (R)	
EF1α	elongation factor 1-alpha	ACGGCGGATATCCTTAACTG (F)	107
		CTGAAGTGAAGTCCGTCGAA (R)	

^1^ F: Forward primer; R: Reverse primer.
